# The Role of Anti-Phosphatidylserine/Prothrombin Antibodies in Thrombotic Manifestations of Systemic Lupus Erythematosus Patients

**DOI:** 10.3389/fneur.2013.00066

**Published:** 2013-06-04

**Authors:** Konstantinos Tselios, Alexandros Sarantopoulos, Ioannis Gkougkourellas, Panagiota Boura

**Affiliations:** ^1^Clinical Immunology Unit, Second Department of Internal Medicine, Hippokration General Hospital, Aristotle University of ThessalonikiThessaloniki, Greece

Sir,

In their recent article, published in *Frontiers in Neurology*, Mullen et al. (2012) demonstrated that anti-phosphatidylserine/prothrombin (PS/PT) antibodies are independently associated with stroke or death in non-antiphospholipid syndrome (APS) patients with transient ischemic attacks (TIA). Despite thorough statistics, the authors mention that these findings should be cautiously interpreted, since only two patients had positive IgG anti-PS/PT antibodies and a clinical event. We have recently assessed the influence of this antiphospholipid antibody (aPL) specificity in the clinical phenotype of systemic lupus erythematosus (SLE) patients.

In this regard, we evaluated 35 lupus patients (33 females/2 males, mean age 37.5 ± 8.8 years, mean disease duration 103.8 ± 55.1 months) and one female non-lupus patient (33 years old), according to a previous history of thrombotic complications (arterial and/or venous thrombosis, spontaneous abortions) and other aPL-related manifestations, such as thrombocytopenia and livedo reticularis. Anti-PS/PT antibodies were measured, shortly after the clinical event, by ELISA (AESKULISA, Serin Prothrombin GM, REF 3226). Other aPL specificities, such as anticardiolipin antibodies (ACA), anti-b2GPI antibodies, and lupus anticoagulant (LA) were also assessed.

Positive anti-PS/PT antibodies (>18 U/ml) were detected in 11/35 patients (31.4%), 7 with IgG anti-PS/PT, 1 with IgM anti-PS/PT, and 3 with concomitant IgM and IgG anti-PS/PT. From these patients, seven had a recent history of vascular thrombosis, while the remaining four had other aPL-related symptoms and end-stage renal disease (1/11), recurrent serositis (2/11), and false positive serological reactions for syphilis (1/11) respectively. It should be mentioned that these four patients had the lower titers of anti-PS/PT antibodies (marginally positive). Ten out of 11 patients were simultaneously positive for other aPL, such as ACA and anti-b2GPI antibodies. All patients with thrombosis were premenopausal women and had central nervous system involvement (4/7 multiple infarcts, 1/7 psychosis, 1/7 seizures, and 1/7 posterior reversible leukoencephalopathy syndrome, PRES). Of note, the three latter patients had no visible infarcts in brain MRI but detectable cerebral flow abnormalities in single-photon emission computed tomography (SPECT). None of these patients had traditional atherosclerotic risk factors.

High titers of anti-PS/PT antibodies (IgM and IgG) were also detected in the sole non-lupus patient in three separate cases. This patient manifested cerebrovascular disease (multiple infarcts), in the absence of atherosclerotic risk factors and other aPL. In this case a diagnosis of APS was made, based in the presence of these antibodies.

These findings come in agreement with previous reports demonstrating that the anti-PS/PT antibodies are strongly related to venous and/or arterial thrombotic manifestations in SLE patients and, particularly, cerebral infarctions (Nojima et al., 2006; Nojima et al., 2004). On a pathophysiologic basis, a possible synergistic action of the anti-PS/PT, ACA, and anti-b2GPI antibodies in the induction of ADP-mediated platelet aggregation has been proposed (Nojima et al., 2004). In accordance with these results, Syuto et al. (2009) showed that these antibodies are more frequently (and in higher titers) detected in patients with neuropsychiatric SLE in general.

Our results, although restricted in SLE, confirm the findings of Mullen et al. (2012) that anti-PS/PT antibodies may serve as a surrogate marker for unfavorable outcome in TIA and may represent a subsequent stage in disease evolution in lupus patients. Given that, in many patients, TIA may not become clinically apparent, it could be hypothesized that anti-PS/PT antibodies (present in a lupus patient with quiescent TIA) may lead, over time, to multiple infarcts or to subclinical cerebral flow disturbances, which can predispose to the development of other neuropsychiatric SLE features. The authors also underline that it is difficult to determine if the mechanism behind anti-PS/PT antibodies and cerebrovascular events is primarily thrombotic or atherosclerotic. In our study, where all patients were premenopausal women and had no traditional atherosclerotic risk factors, it could be assumed that thrombosis, rather than atherosclerosis, represents the main pathophysiologic mechanism.

In conclusion, anti-PS/PT antibodies seem to be strongly related to ischemic/thrombotic cerebrovascular events. Evaluation of their predictive ability and stratification of patients with TIA, in large scale prospective studies, is warranted.
